# Properties of Ozone-Oxidized Tapioca Starch and Its Use in Coating of Fried Peanuts

**DOI:** 10.3390/molecules26206281

**Published:** 2021-10-17

**Authors:** Yudi Pranoto, Brigitta Laksmi Paramita, Muhammad Nur Cahyanto, Soottawat Benjakul

**Affiliations:** 1 Department of Food and Agricultural Products Technology, Faculty of Agricultural Technology, Universitas Gadjah Mada, Jl. Flora No. 1, Bulaksumur, Yogyakarta 55281, Indonesia; brigittalaksmi@yahoo.com (B.L.P.); mn_cahyanto@ugm.ac.id (M.N.C.); 2 Department of Food Technology, Faculty of Agro-Industry, Prince of Songkhla University, Hat Yai 90112, Songkhla, Thailand; soottawat.b@psu.ac.th

**Keywords:** tapioca, oxidation, ozone, time, pH, frying expansion, coated peanut

## Abstract

Oxidation of tapioca via ozone oxidation was carried out under different conditions in comparison with H_2_O_2_. The impact of ozonation on physicochemical properties of tapioca was studied and fried peanuts coated with different tapioca were characterized. Different ozone oxidation times (10, 20, and 30 min) and various pH values (5, 7, and 9) were used for tapioca modification. Tapioca oxidized by ozone for 20 min at pH 7 had higher swelling power (SP), water holding capacity (WHC), oil holding capacity (OHC), and viscosity than the native counterpart (*P* < 0.05). This coincided with the higher carbonyl and carboxyl contents (*P* < 0.05). The highest frying expansion (FE) with the lowest hardness was attained for fried peanut coated with tapioca oxidized under the aforementioned condition. Therefore, oxidation of tapioca using ozone under optimal conditions could be a potential means to improve frying expansion as well as the crispiness of the fried coated peanuts.

## 1. Introduction

Cassava (*Manihot esculenta*) is the potential carbohydrate, apart from corn and rice. Cassava is usually processed into cassava starch, known as tapioca. Tapioca has been used for coating of several fried products. Coated peanuts are popular fried snacks made from peanuts coated with tapioca. The crispness is the most important properties of this fried snack, and it has correlation with the expansion properties of the coating material. Tapioca in its native state has limited expansion and crispiness [1]. Therefore, it should be modified to obtain the desired characteristics.

Oxidation is one of chemical modifications of the starch, which is able to improve its expansion [2,3]. Oxidation process involves alteration of hydroxyl groups to carbonyl (CNB) groups and subsequently to carboxyl (CBX) groups. This phenomenon mainly takes place at C-2 and C-3 positions [4,5]. CBX groups have a crucial role in the expansion improvement [6]. Generally, sodium hypochlorite as well as hydrogen peroxide have been used for starch oxidation, and their residues can be found in food products [7]. Ozone is one of alternative oxidizing agents and is considered safe for food product because it is completely decomposed [6]. OH radical resulted from ozone plays a role in the oxidation process, causing partial depolymerization or degradation of the starch by breaking down α 1,4-glycosidic linkages into shorter fragments, as well as oxidizing OH group of the fragmented starches into CBN and subsequently CBX. Nevertheless, pro-oxidant effect of ozone has been documented [8,9]. Ozonation with different ozone generation times (OGT) showed structural and properties changes on corn, sago, and cassava starches [6,10]. Klein et al. [11] also demonstrated that ozonation with different pH caused the changes in properties of tapioca. The highest CBX and CBN contents were obtained at pH 9.5.

Although the properties of ozone-oxidized starch have been reported, there is limited information on the properties of fried products using oxidized tapioca as a coating material (shell). Therefore, the study aimed to characterize the influence of different ozone oxidation times and pH values on the physicochemical properties of tapioca and properties of fried coated peanuts.

## 2. Results and Discussion

### 2.1. Characteristics and Properties of Tapioca as Influenced by Ozone Oxidation under Different Conditions

#### 2.1.1. Carbonyl (CBN) and Carboxyl (CBX) Contents

CBN and CBX contents of ozone-oxidized tapioca are shown in Table 1. Both CBN and CBX contents of ozone-oxidized tapioca were greater than those of native counterpart. Ozone in aqueous solution is generally decomposed into OH radical, which increases as pH solution increases [12]. OH radical resulted from ozone plays a role in oxidation process, causing partial depolymerization of the starch by breaking down α 1,4-glycosidic linkages into shorter fragment, oxidizing and converting OH group of the fragmented starches into CBN and subsequently CBX groups [5]. Thus, the numbers of CBN and CBX groups in oxidized tapioca reflect the degree of oxidation, taken place primarily at the hydroxyl groups at C-2, C-3, and C-6 positions [13]. Oxidation with ozone affected the CBN contents of the tapioca. Tapioca oxidized for 20 min at pH 7 resulted in the highest CBN content. Thus, both pH and oxidation time played a profound role in oxidation of tapioca.

Similarly, different pH condition significantly affected CBX content of ozone-oxidized tapioca. CBX content increased as pH increased up to pH 7, and there was no change at pH 9. This phenomenon suggested that the oxidizing species (free radicals and nascent oxygen) were excessive and could mutually destroy themselves by coupling [14]. Decomposition of ozone becomes faster with the increase of pH and the abundance of OH radicals [15]. Overall, CBX contents of tapioca oxidized by ozone were higher than that of tapioca oxidized by H_2_O_2_ (*P* < 0.05). It indicted that oxidation process with ozone at this condition was more intensive compared to H_2_O_2_.

#### 2.1.2. Amylose Content

Amylose content of ozone-oxidized tapioca is shown in Table 1. Among all the samples, native tapioca showed the lowest amylose content (*P* < 0.05). Oxidation process positively affected amylose content of tapioca (*P* > 0.05). However, the sample oxidized with H_2_O_2_ had higher amylose content than ozone-oxidized tapioca (*P* < 0.05). Sandhu et al. [8] documented that amylose content of oxidized wheat starch oxidized with ozone increased from 28.9 to 34.4%, compared to the native counterpart. The increase of amylose content is because of depolymerization of amylose and amylopectin during the oxidation process into shorter chains and is identified as amylose [16]. According to Wang and Wang [5], amylose is more susceptible against depolymerization than amylopectin. In addition, oxidation mostly took place in the amorphous regions of starch granules where most are amylose [13]. Depolymerization in starch granules increased the amount of hydroxyl groups in the granules where the hydroxyl group also has a role in the hydration ability of starch granules [17]. According to Chan et al. [10], depolymerized starch granules enabled it to absorb water easier.

#### 2.1.3. Swelling Power (SP) and Solubility

SP and solubility of the starches are shown in Table 2. Overall, oxidation time affected SP rather than the pH; the longer oxidation time led to higher SP. Also, most of ozone-oxidized tapioca had lower SP than that oxidized with H_2_O_2_ for the exception that the highest SP was obtained for the sample oxidized with ozone for 30 min at pH 9. This was possibly due to degradation of starch granule and the presence of carboxyl content, which could improve hydration ability of starch [10]. The highest SP of sample oxidized by ozone for 30 min could be related to effective oxidation process. Hydration ability of starch involves hydroxyl groups in amylose and amylopectin chain and hydrophilic properties of CBX groups formed from oxidation process [18].

Ozone-oxidized tapioca had higher solubility than their native counterpart. A similar result was noticeable with H_2_O_2_-oxidized tapioca, which showed the highest solubility. The higher solubility can be related to the degradation of starch granules. Degradation of starch granules also impairs structure of starch granule as indicated by the improved solubility [10,19]. Therefore, oxidation of tapioca could increase both SP and solubility.

#### 2.1.4. Water Holding Capacity (WHC) and Oil Holding Capacity (OHC)

WHC and OHC are shown in Table 2. Ozone-oxidation increased WHC of tapioca, compared to native counterpart. However, there was no difference in WHC among all ozone-oxidized samples as well as H_2_O_2_-oxidzed sample (*P* > 0.05). Generally, the higher WHC is associated with the breaking of the glycosidic bonds in starch granules and the formation of functional groups during oxidation process, which can cause erosion in amorphous region [20]. In addition, repulsive interaction between functional groups of oxidized starch can facilitate water to enter the starch granules, resulting in higher WHC [21].

A bit different from WHC, OHC of all oxidized starches were lower than the native one. Lower OHC of oxidized starch attained was because of cross-linking induced by slight oxidation [5,11]. Among ozone-oxidized tapioca, OHC increased as ozone oxidation time increased up to 20 min, while 30 min had no further impact (*P* > 0.05). The higher OHC is caused by the erosion of amorphous regions and functional groups formation in starch granule, which is expected to facilitate oil to penetrate into the starch granules during frying [20].

#### 2.1.5. Pasting Properties

**Figure 1:**
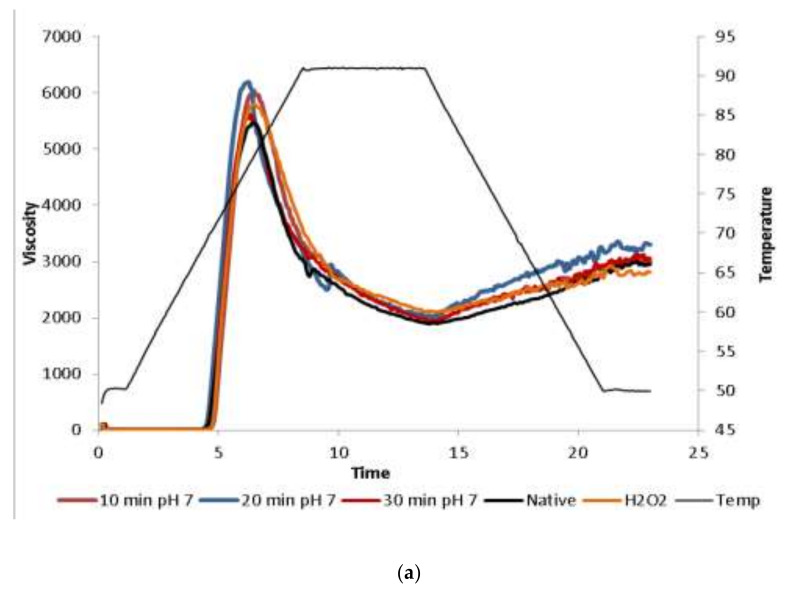

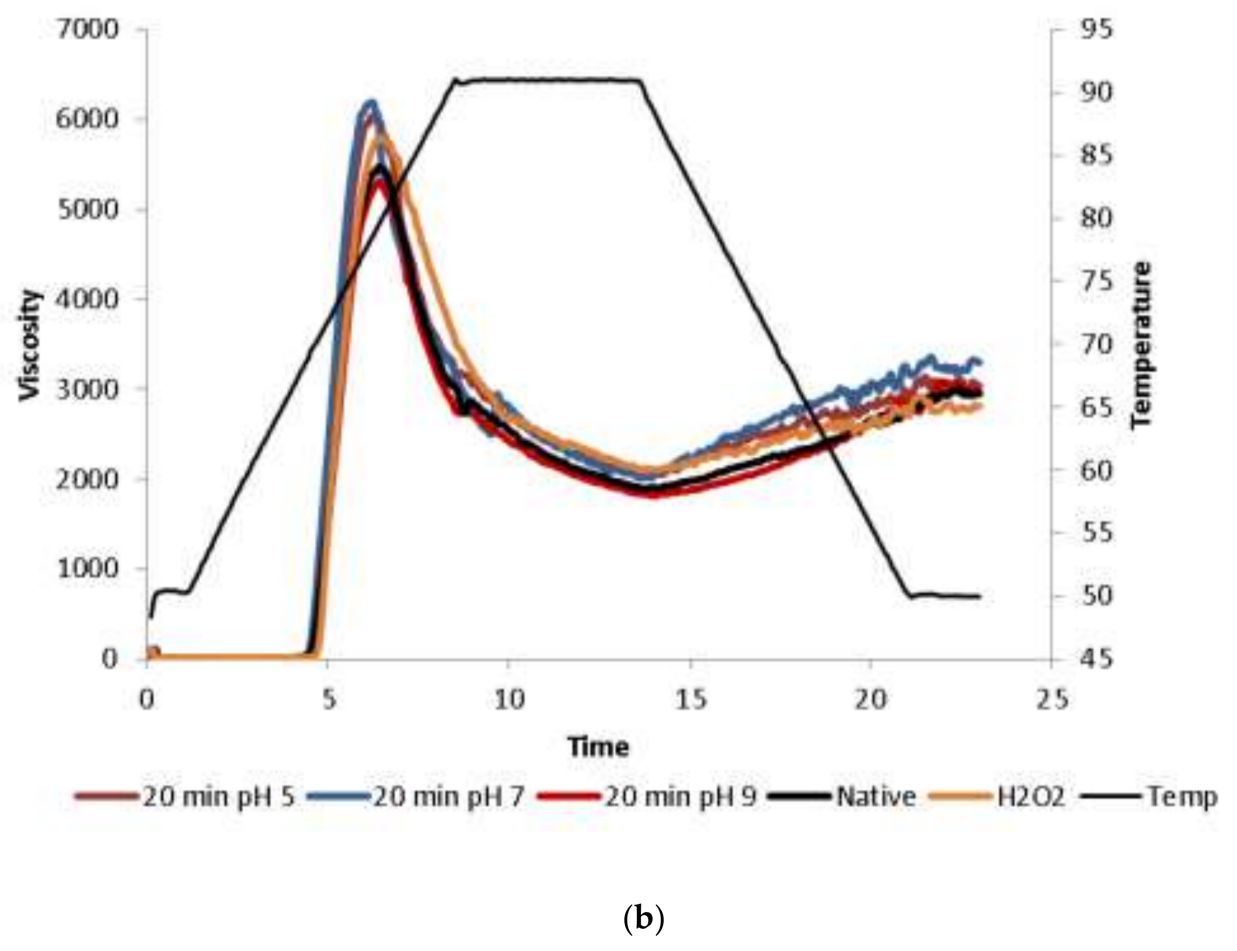
RVA curve of ozone-oxidized tapioca with different oxidation time at pH 7 (**a**) and different pH for 20 min (**b**) compared with native and H_2_O_2-_oxidized tapioca.

RVA curve of pasting properties of native and ozone-oxidized tapioca in different oxidation times at the selected pH 7 and varying pH conditions of selected 20 min are illustrated in Figure 1. Ozone-oxidized tapioca showed higher viscosity than native tapioca. Generally, oxidized starch has lower viscosity than the native counterpart, possibly due to degradation of starch granule induced by the oxidation process [10]. However, tapioca oxidized using ozone for 20 min at pH 7 had the highest final viscosity compared to other treatments. The longer oxidation time yielded the tapioca with higher breakdown and peak viscosity (Figure 1).

The higher viscosity was possibly mediated by hemiacetal cross-linking between depolymerized starch chain due to low level of oxidants [11]. Hemiacetal cross-linking stabilizes swollen granules and circumvent minor depolymerization. Aldehyde groups formed due to oxidation process can form hemiacetal cross-links [5]. Farley and Hixon [22] also suggested that higher viscosity was possibly from higher swelling related with higher hydration mediated by CBX groups. Although the starch oxidized by ozone for 20 min at pH 7 showed the highest final viscosity, this treatment also showed the highest breakdown. High breakdown indicates low viscosity after gelatinization and it can improve bubble growth and generate higher expansion. Nevertheless, there is no specific explanation of this phenomenon [2].

At the same time (20 min), for oxidation by ozone, no changes in pasting temperature were noticeable (*P* > 0.05). Nevertheless, the highest peak viscosity, breakdown, setback and final viscosity were achieved at pH 7 (Figure 1).

### 2.2. Characteristics and Properties of Fried Peanut Coated with Ozone-Oxidized Tapioca

**Figure 2:**
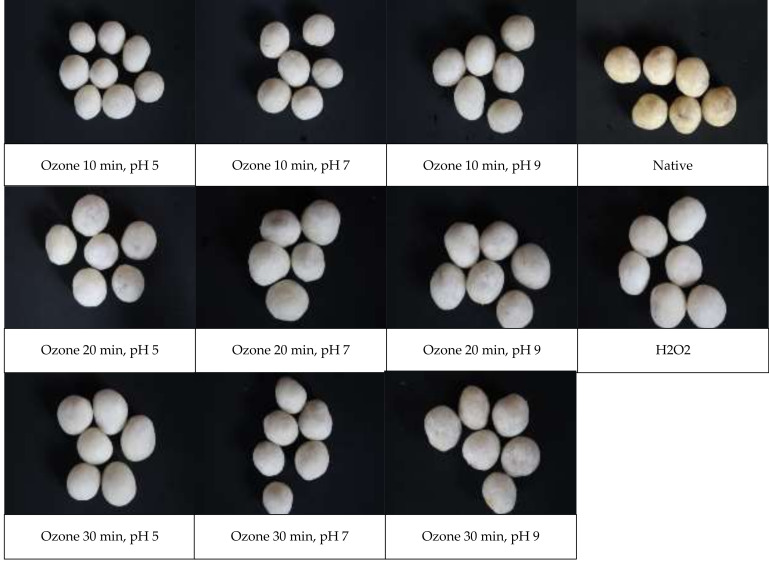
Samples of fried peanuts coated with ozone-oxidized tapioca compared to native and H_2_O_2_-oxidized tapioca.

The fried peanuts coated with various tapioca were seen in Figure 2. The fried peanuts show different appearance, color, and expansion due to frying. Therefore, some relevant properties of the fried product are subsequently evaluated.

#### 2.2.1. Frying Expansion (FE)

**Figure 3:**
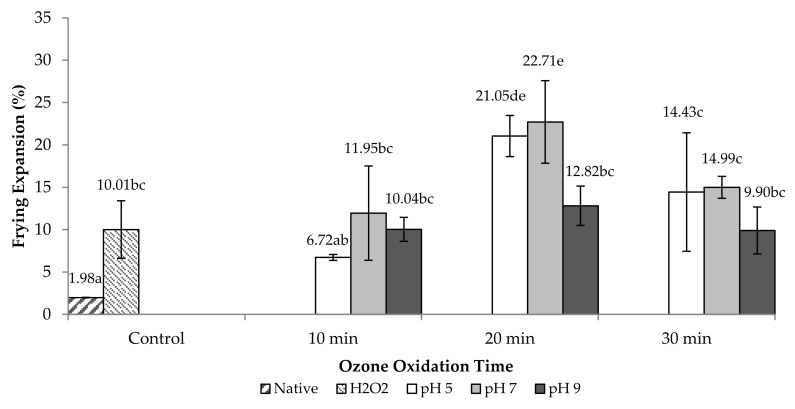
Frying expansion of ozone-oxidized tapioca-coated peanuts compared with native and H_2_O_2_-oxidized tapioca. Bars represent the standard deviation (*n* = 3). Different uppercase letters on the bars indicate the significant difference (*P* < 0.05).

Depolymerization of chains in starch granules due to oxidation can increase frying expansion due to the looser structure. FE property of fried coated peanut is depicted in Figure 3. In general, oxidation of starch led to higher FE of fried products, as is seen in Figure 2 as well. Ozone-oxidized tapioca had higher expansion than native and H_2_O_2_-oxidized tapioca. Ozone-oxidized tapioca (20 min and pH 5, pH 7) showed the highest FE (*P* < 0.05) compared to the others. FE occurred, mostly caused by the vapor pressure of trapped water, which is increased when the temperature is augmented, thus inducing bubble growth. The more water trapped can more likely enhance the more bubble growth in the dough, therefore resulting in higher expansion [2]. Priadi [23] reported that swelling power can be related to expansion. Higher SP can improve expansion due to higher hydration ability of starch granule.

Trapped water could be related to hydrophilic ability of the starch granule, in which CBX groups and degradation of the starch granule played a profound role, whereas hydrophilic ability of the starch granule is associated with amylose molecules, which is the major component of the amorphous region [17,18]. The higher breakdown and good heat transfer can also induce bubble growth [2,24]. It was noted that ozone-oxidized tapioca also had the increased water holding capacity (WHC) (Table 2). This could help expand the starch granule during frying. However, Nurul et al. [25] reported that product with higher OHC showed higher expansion. High oil absorption allows good heat transfer, which can generate bubble growth, resulting in higher expansion during frying [2]. In addition, expansion is also governed by several factors such as hydration ability of starch and starch viscosity after gelatinization.

#### 2.2.2. Hardness

**Figure 4:**
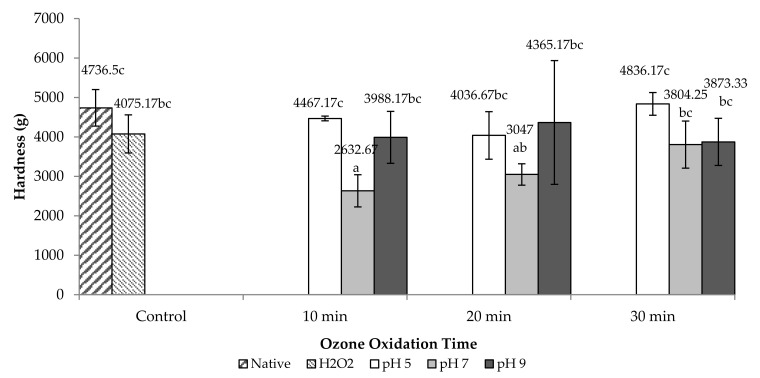
Hardness of ozone-oxidized tapioca-coated peanuts compared with native and H_2_O_2_-oxidized tapioca. Bars represent the standard deviation (*n* = 3). Different uppercase letters on the bars indicate the significant difference (*P* < 0.05).

Hardness of fried coated peanut is shown in Figure 4. Ozone-oxidized tapioca coated on fried peanut had lower hardness than native and H_2_O_2_-oxidized tapioca in fried peanut. However, the lower hardness (*P* < 0.05) was found with the oxidation time of 10 and 20 min at pH 7. Nurul et al. [23] reported that low hardness value indicated a high degree of crispiness. The pH 7 treatment had lower hardness value than other pH treatments (Figure 3), where the pH 7 treatment also had a higher expansion than pH 5 and 9 (Figure 2). Ngadi et al. [26] suggested that crunchy textures can be related to fragile porous structures formed by rapid evaporation of water in the product and an increase in sufficient internal pressure for matrix formation.

#### 2.2.3. Microscopic Structure

**Figure 5:**
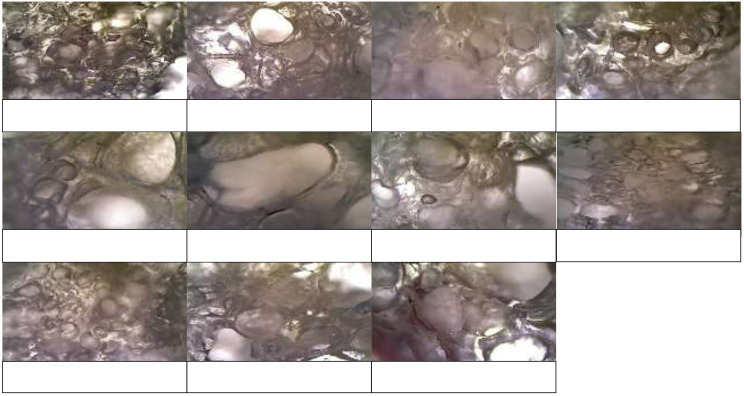
Microscopic structure of fried peanut coated with ozone-oxidized tapioca compared to native and H_2_O_2_-oxidized tapioca.

The microscopic structures of fried peanuts coated with tapioca are illustrated in Figure 5. Ozone-oxidized tapioca had larger pores with thinner partition than the native counterpart. It could be related to the lower hardness value and more crunchy texture. Ozone-oxidized tapioca (oxidation time 20 min and pH 7) had larger pores, compared to that oxidized using H_2_O_2_. Porous structure is associated with the crispy texture. The product with smaller pores had higher hardness value. Porosity of product can be examined by the expansion ratio that is in accordance with frying expansion characteristics [27].

During frying, the water vapor pressure will induce the formation of porous structures in the product [26]. Tapioca oxidized with ozone for 20 min at pH 7 had larger pores than those oxidized for 10 and 30 min. Also, at the same oxidation time (20 min), tapioca oxidized at pH 7 rendered the large pore better than pH 5 and pH 9. This was also supported by the higher expansion in this treatment (ozone oxidation for 20 min at pH 7) due to high hydrophilic ability of starch granules and high heat transfer during frying.

## 3. Materials and Methods

### 3.1. Materials

Native cassava starch/tapioca and unshelled peanuts (the medium size of average diameter of 7 mm) were procured from the local market in the city of Yogyakarta, Indonesia. All the reagents were of analytical grade.

### 3.2. Preparation of Ozone-Oxidized Tapioca

Ozone-oxidized tapioca was prepared as tailored by Satmalawati [28] with a slight modification. Tapioca was firstly mixed with deionized water at 1:8 ratio (*w*/*v*) and the mixture were adjusted to pH 5, 7, and 9 with 0.01 N NaOH or 0.05% citric acid. The suspension was bubbled with ozone (0.18–0.41 g ozone/h) for 10, 20, and 30 min. The suspension was washed until neutral pH was obtained. The oxidized tapioca was then dried at 50 °C for 24 h using an oven dryer until the moisture content of 11% was gained. Ozone-oxidized tapioca was then sieved with 80 mesh sieve. Tapioca oxidized with H_2_O_2_ was also used as comparison. Oxidized tapioca with H_2_O_2_ was prepared by mixing tapioca suspension with 0.1% H_2_O_2_ at pH 7 for 20 min. All tapioca samples including native tapioca were subjected to analyses.

#### 3.2.1. Carbonyl (CBN) Content

CBN content was measured as detailed by Kuakpetoon and Wang [13]. In a total of 100 mL of distilled water placed in a 500 mL flask, starch (4 g) was added and stirred. The resulting suspension was placed in a boiling water bath for 20 min. Thereafter, the gelatinized sample was cooled to 40 °C and pH was subsequently adjusted to pH 3.2 using 0.1 N HCl. After that, hydroxylamine reagent (15 mL) was added. The flask was stoppered and incubated in a water bath (40 °C) with gentle stirring for 4 h. The excessive hydroxylamine was quantified by rapidly titrating the reaction mixture with standardized 0.1 N HCl to obtain pH of 3.2. A blank was prepared in the same manner, except only hydroxylamine reagent was used. CBN content was calculated as follows:

(1) $$CBN\;content\;\left(\%\right)\;=\frac{\left(Vb-Vs\right)\;\times \;N\;\times \;0.028\;\times \;100}{W}$$

where *Vb* is mL of HCl used for the blank; *Vs* is mL of HCl used for sample; *N* is HCl concentration (*N*), and *W* is sample weight (g, dry basis).

#### 3.2.2. Carboxyl (CBX) Content

CBX content was measured as reported by Sangseethong et al. [29] with a slight modification. Starch sample (5 g) was stirred in 25 mL of 0.1 M HCl for 30 min, followed by filtration using a filter paper. The samples were washed with distilled water until no chloride ions were detected. The filtered cake was mixed with distilled water to obtain the final volume of 300 mL in a 600 mL beaker. Slurry was then subjected to heating in a boiling water bath with continuous stirring for 15 min, in which gelatinization was complete. Immediately, the gelatinized sample was titrated with 0.1 M NaOH, in which phenolphthalein was used as an indicator. A blank was prepared using native tapioca starch. CBX content was calculated as follows:

(2) $$CBX\;content\;\left(\%\right)\;=\frac{\left(Vs-Vb\right)\;\times \;N\;\times \;0.045\;\times \;100}{W}$$

where *Vb* is mL of NaOH used for the blank; *Vs* is mL of NaOH for sample; *N* is NaO concentration; and *W* is sample weight (g, dry basis).

#### 3.2.3. Amylose Content

Amylose content was measured according to the method of AOAC [30]. The amylose content was calculated from a standard curve prepared using a pure amylose with the concentration of 0.4, 0.8, 1.2, 1.6, and 2.0 % and was expressed as percentage.

#### 3.2.4. Swelling Power and Solubility

Swelling power and solubility were determined following the method of Adebowale et al. [20]. A starch sample (1.0 g) was accurately weighed and placed in a clear dried test tube and reweighed (W1). To the tube, 50 mL of distilled water were added, and the mixture was heated at 95 °C for 30 min, followed by cooling to 30 °C and centrifugation (1700× *g*, 15 min). The supernatant (5 mL) was dried at 110 °C using an oven dryer (Model UN55 Plus 53L, MEMMERT GmbH + Co. KG, Schwabach, Germany) until the constant weight was achieved, representing starch solubilized in water. Solubility was expressed as g per 100 g of starch on a dry weight basis.

The pellet obtained after centrifugation was transferred to the clean dried test tube and weighed (*W*2). Swelling power content was calculated as follows:

(3) $$Swelling\;Power\;\left(\%\right)\;=\frac{W2-W1}{Sample\;weight\;\left(g\right)}$$

#### 3.2.5. Water Holding Capacity (WHC) and Oil Holding Capacity (OHC)

WHC and OHC were examined as per the method of Uzomah and Ibe [21]. Water or oil (10 mL) was mixed with 1 g of the sample in a centrifuge tube of known weight (sample weight + tube = W1). The mixture was left for 30 min prior to centrifugation (3500× *g*, 15 min) and the supernatant was discarded. The tube and the pellet were weighed (W2). WHC and OHC were calculated as follows:

(4) $$WHC\;\left(mL/g\right)\;=\frac{W2-W1}{Sample\;weight\;\left(g\right)}$$

(5) $$OHC\;\left(mL/g\right)\;=\frac{m.oiled-sample\;weight}{Sample\;weight\;\left(g\right)}$$

where *m*. *oiled* is *W*2 minus tube weight.

#### 3.2.6. Pasting Properties

Pasting properties were determined using Rapid Visco Analyzer (RVA 4500, Perten, Sweden). To prepare the sample, 25 mL of distilled water were added into 3 g of sample in a RVA canister and mixed thoroughly. Suspension was subjected to heating at 50 °C for 1 min and heating was continued up to 90 °C for 7 min and 30 s with a rate 5.3 °C/min and hold for 5 min. Temperature was then decreased to reach 50 °C with a rate 5.3 °C/min and hold for 2 min. Pasting curve was then recorded and evaluated for the pasting parameters.

### 3.3. Preparation of Fried Peanut Coated with Ozone-Oxidized Tapioca

The method of Suprapti [31] was followed with a slight modification. Native, H_2_O_2_-oxidized, and ozone-oxidized tapioca starches (5 g) were dispersed and heated in 20 mL of distilled water until gelatinization took place. Gel was then mixed with 17.5 g of dry tapioca (native or oxidized tapioca). The dough was then used to coat unshelled ground peanut with thickness of 2 mm and was subsequently fried in palm oil at 170 °C for 15 min. The products obtained were subjected to analyses.

#### 3.3.1. Frying Expansion (FE)

FE of fried coated peanut was determined following the method of Nguyen et al. [32]. Coated peanut samples (10 pcs) were placed in a measuring glass assisted with bread crumb and the volume was recorded (*V*1). After frying, samples were placed again in a measuring glass and the final volume was recorded (*V*2). FE was calculated as follows:


(6) $$\%\;FE\;=\frac{V2-V1}{V1}\;\times \;100\%$$

#### 3.3.2. Hardness

Hardness of fried coated peanut was examined as detailed by Nguyen et al. [32]. Hardness of coated peanut was determined using TA-XT Plus Texture Analyzer (Stable Micro Systems; Godalming, UK) software Texture Exponent 32. Maximum force in g was recorded as the hardness.

#### 3.3.3. Microstructures

Microstructures of fried coated peanut were visualized as per the method of Saeleaw and Schleining [33] using a microscope (OLYMPUS CX21LED, Olympus, Japan). Cross section of fried tapioca starch coating was viewed at 10× magnification.

### 3.4. Statistical Analysis

The data from each treatment were subjected to analysis of variance (ANOVA) and the significant differences among means were determined by *Duncan Multiple’s Range Test* (DMRT) at 95% confidence level using SPSS software version 17.0 (SPSS Inc., Chicago, IL, USA).

## 4. Conclusions

Oxidation process using ozone for different oxidation times and pH conditions significantly affected physicochemical properties of tapioca. SP, viscosity, WHC, and FE increased due to oxidation by ozone, and optimum condition was found at pH 7 for 20 min. This was concomitant with the increased CBN and CBX contents, which were associated with crispy texture or less hardness of fried coated peanut. Tapioca oxidized by ozone under the optimal condition yielded the highest frying expansion of fried coated peanut. It could be related to hydration ability of starch granule, in which CBX groups and degradation of starch granule were augmented.

## Figures and Tables

**Table 1:** Carbonyl, carboxyl, and amylose content of ozone-oxidized tapioca compared to native and H_2_O_2_-oxidized tapioca.

Oxidation Condition		Carbonyl (%) ^1^	Carboxyl (%) ^1^	Amylose (%) ^1^
Oxidation Time pH				
Native		0.007 ± 0.011 ^a^	0.0000 ± 0.0015 ^a^	32.54 ± 3.96 ^a^
H_2_O_2_		0.270 ± 0.03 ^b^	0.0036 ± 0.0037 ^ab^	44.34 ± 1.29 ^d^
10 min	pH 5	0.309 ± 0.03 ^bc^	0.0070 ± 0.0018 ^bc^	35.65 ± 0.97 ^ab^
	pH 7	0.353 ± 0.04 ^bc^	0.0088 ± 0.0030 ^c^	38.63 ± 0.70 ^bc^
	pH 9	0.241 ± 0.03 ^b^	0.0063 ± 0.0001 ^bc^	37.54 ± 1.11 ^b^
20 min	pH 5	0.353 ± 0.06 ^bc^	0.0061 ± 0.0026 ^bc^	37.92 ± 1.74 ^bc^
	pH 7	0.390 ± 0.10 ^c^	0.0103 ± 0.0021 ^c^	41.38 ± 1.43 ^cd^
	pH 9	0.323 ± 0.02 ^bc^	0.0083 ± 0.0029 ^c^	37.80 ± 1.19 ^b^
30 min	pH 5	0.314 ± 0.04 ^bc^	0.0071 ± 0.0015 ^bc^	39.07 ± 1.94 ^bc^
	pH 7	0.300 ± 0.09 ^bc^	0.0087 ± 0.0015 ^c^	38.41 ± 2.94 ^bc^
	pH 9	0.269 ± 0.01 ^b^	0.0092 ± 0.0033 ^c^	38.11 ± 0.25 ^bc^

^1^ Mean ± standard deviation. Values with different superscript letters in the same column indicate significant difference (*P* < 0.05).

**Table 2:** Swelling power, solubility, WHC, and OHC ozone-oxidized tapioca compared to native and H_2_O_2_-oxidized tapioca.

Oxidation Condition		Swelling Power (%) ^1^	Solubility (%) ^1^	WHC (%) ^1^	OHC (%) ^1^
Oxidation Time pH					
Native		13.27 ± 0.78 ^a^	0.18 ± 0.01 ^a^	0.76 ± 0.01 ^a^	1.20 ± 0.04 ^d^
H_2_O_2_		21.12 ±1.57 ^c^	0.34 ± 0.08 ^c^	0.97 ± 0.02 ^b^	0.76 ± 0.01 ^a^
10 min	pH 5	16.54 ± 1.78 ^ab^	0.20 ± 0.02 ^a^	0.98 ± 0.03 ^b^	0.88 ± 0.03 ^bc^
	pH 7	17.10 ± 1.36 ^b^	0.25 ± 0.02 ^bc^	0.89 ± 0.08 ^b^	0.84 ± 0.05 ^b^
	pH 9	15.70 ± 1.33 ^ab^	0.23 ± 0.06 ^ab^	0.91 ± 0.06 ^b^	0.87 ± 0.01 ^bc^
20 min	pH 5	18.07 ± 3.25 ^bc^	0.23 ± 0.01 ^ab^	0.99 ± 0.05 ^b^	0.95 ± 0.02 ^c^
	pH 7	17.95 ± 1.22 ^bc^	0.27 ± 0.06 ^bc^	0.95 ± 0.03 ^b^	0.94 ± 0.05 ^c^
	pH 9	16.54 ± 1.41 ^ab^	0.25 ± 0.08 ^bc^	0.92 ± 0.08 ^b^	0.91 ± 0.08 ^bc^
30 min	pH 5	17.00 ± 1.32 ^b^	0.19 ± 0.04 ^a^	0.92 ± 0.10 ^b^	0.91 ± 0.06 ^bc^
	pH 7	17.86 ± 1.68 ^bc^	0.20 ± 0.01 ^a^	0.92 ± 0.03 ^b^	0.86 ± 0.02 ^bc^
	pH 9	24.60 ± 2.73 ^d^	0.20 ± 0.02 ^a^	0.89 ± 0.04 ^b^	0.91 ± 0.03 ^bc^

^1^ Mean ± standard deviation. Values with different superscript letters in the same column indicate significant difference (*P* < 0.05).

## Data Availability

The data presented in this study are available on request from the corresponding author. The data are not publicly available due to ethical restriction and the intellectual property issue.

## Sample Availability

Samples of the oxidized tapioca starch are available from the authors.

## References

[1] Franco C.M.L., Ogawa C., Rabachini T., Rocha T.D.S., Cereda M.P., Jane J.-I. (2010). Effect of lactic acid and UV irradiation on the cassava and corn starches. Braz. Arch. Biol. Technol..

[2] Bertolini A.C., Mestres C., Lourdin D., Valle G.D., Colonna P. (2001). Relationship between thermomechanical properties and baking expansion of sour cassava starch (*Polvilho azedo*). J. Sci. Food Agric..

[3] Demiate I.M., Dupuy N., Huvenne J.P., Cereda M.P., Wosiacki G. (2000). Relationship between baking behavior of modified cassava starches and starch chemical structure determined by FTIR spectroscopy. Carbohydr. Polym..

[4] Tharanathan R.N. (2005). Starch-value addition by modification. Crit. Rev. Food Sci. Nutr. Polym..

[5] Wang Y.-J., Wang L. (2003). Physicochemical properties of common and waxy corn starches oxidized by different levels of sodium hypochlorite. Carbohydr. Polym..

[6] Chan H.T., Leh C.P., Bhat R., Senan C., Williams P.A., Karim A.A. (2011). Molecular structure, rheological and thermal characteristics of ozone-oxidized starch. Food Chem..

[7] Dias A.R.G., Zavareze E.d.R., Elias M.C., Helbig E., da Silva D.O., Ciacco C.F. (2011). Pasting, expansion and textural properties of fermented cassava starch oxidised with sodium hypochlorite. Carbohydr. Polym..

[8] Sandhu H.P.S., Manthey F.A., Simsek S. (2012). Ozone gas affects physical and chemical properties of wheat (*Triticum aestivum* L.) starch. Carbohydr. Polym..

[9] Oladebeye A.O., Oshodi A.A., Amoo I.A., Karim A.A. (2013). Functional, thermal and molecular behaviours of ozone-oxidised cocoyam and yam starches. Food Chem..

[10] Chan H.T., Bhat R., Karim A.A. (2009). Physicochemical and functional properties of ozone-oxidized starch. J. Agric. Food Chem..

[11] Klein B., Vanier N.L., Moomand K., Pinto V.Z., Colussi R., da Rosa Zavareze E., Dias A.R.G. (2014). Ozone oxidation of cassava starch in aqueous solution at different pH. Food Chem..

[12] Rodríguez A., Rosal R., Perdigón-Melón J.A., Mezcua M., Agüera A., Hernando M.D., Letón P., Fernández-Alba A.R., García-Calvo E., Barceló D., Kostianoy A.G. (2008). Ozone based technologies in water and wastewater treatment. Handbook of Environmental Chemistry, Volume 5: Water Pollution.

[13] Kuakpetoon D., Wang Y.-J. (2001). Characterization of different starches oxidized by hypochlorite. Starke.

[14] El-Sheikh M.A., Ramadan M.A., El-Shafie A. (2010). Photo-oxidation of rice starch. Part I: Using hydrogen peroxide. Carbohydr. Polym..

[15] Eriksson M. (2005). Ozone Chemistry in Aqueous Solution. Ph.D. Thesis.

[16] Kuakpetoon D., Wang Y.-J. (2006). Structural characteristics and physicochemical properties of oxidized corn starches varying in amylose content. Carbohydr. Res..

[17] Dewi A.M.P. (2011). Tapioca Oxidation with Hydrogen Peroxide and UV C Irradiation Catalyst, and the Application for Edible Film (Indonesian). Master’s Thesis.

[18] Lee J.S., Kumar R.N., Rozman H.D., Azemi B.M.N. (2005). Pasting, Swelling, and Solubility Properties of UV initiated Starch-graft-Poly(AA). Food Chem..

[19] Adebowale K.O., Adeniyi Afolabi T., Lawal O.S. (2002). Isolation, chemical modification and physicochemical characterisation of Bambarra groundnut (*Voandzeia subterranean*) starch and flour. Food Chem..

[20] Uzomah A., Ibe C. (2011). The functional properties, pasting and baking behaviour of chemically modified sour cassava starches. Afr. J. Food Sci..

[21] Lawal O.S., Adebowale K.O., Ogunsanwo B.M., Barba L.L., Ilo N.S. (2005). Oxidized and acid thinned starch derivatives of hybrid maize: Functional characteristics, wide-angle X-ray diffractometry and thermal properties. Int. J. Biol. Macromol..

[22] Farley F.F., Hixon R.M. (1942). Oxidation of raw starch granules by electrolysis in alkaline sodium chloride solution. Ind. Eng. Chem..

[23] Priadi G. (2013). Effect of Oxidation of Shredded Acid Cassava with Hidrogen Peroxide and UV Catalyst in a Tumbler on the Baking Expansion (Indonesian). Master’s Thesis.

[24] Dana D., Saguy I.S. (2001). Frying of nutritious foods: Obstacles and feasibility. Food Sci. Technol. Res..

[25] Nurul H., Boni I., Noryati I. (2009). The effect of different ratios of Dory Fish to tapioca flour on the linear expansion, oil absorption, colour and hardness of fish crackers. Int. Food. Res. J..

[26] Ngadi M., Adedeji A.A., Kassama L., Sahin S., Summu S.G. (2008). Microstructural changes during frying of foods. Advances in Deep-Frying of Foods.

[27] Tsukakoshi Y., Naito S., Ishida N. (2008). Fracture intermittency during a puncture test of cereal snacks and its relation to porous structure. Food Res. Int..

[28] Satmalawati M.M.E.M. (2011). Characterization of Oxidized Tapioca at Varying Dissolved Ozon and Starch Slurry Concentration (Indonesian). Master’s Thesis.

[29] Sangseethong K., Termvejsayanon N., Sriroth K. (2010). Characterization of physicochemical properties of hypochlorite- and peroxide-oxidized cassava starches. Carbohydr. Polym..

[30] AOAC (1984). Official Methods of Analysis.

[31] Suprapti M.L. (2005). Tapioca Flour: Making and its utilization (Indonesian).

[32] Nguyen T.T., Le T.Q., Songsermpong S. (2013). Shrimp Cassava Cracker Puffed by Microwave Technique: Effect of Moisture and Oil Content on Some Physical Characteristics. Witthayasan Kasetsat.

[33] Saeleaw M., Schleining G. (2011). Effect of frying parameters on crispiness and sound emission of cassava crackers. J. Food Eng..

